# Dual-section versus conventional archwire for en-masse retraction of anterior teeth with direct skeletal anchorage: a finite element analysis

**DOI:** 10.1186/s12903-021-01443-0

**Published:** 2021-02-25

**Authors:** Ryo Hamanaka, Daniele Cantarella, Luca Lombardo, Lorena Karanxha, Massimo Del Fabbro, Giuseppe Siciliani, Noriaki Yoshida

**Affiliations:** 1 Department of Orthodontics and Dentofacial Orthopedics, Nagasaki University Graduate School of Biomedical Sciences, 1-7-1 Sakamoto, Nagasaki, 852-8588 Japan; 2 Department of Biomedical, Surgical and Dental Sciences, University of Milan, Via Commenda 10, Milan, Italy; 3 Postgraduate School of Orthodontics, Ferrara University, Via Luigi Borsari 46, Ferrara, Italy; 4 Dental Clinic, IRCCS Orthopedic Institute Galeazzi, Via Riccardo Galeazzi 4, Milan, Italy

**Keywords:** FEM, Finite element method, Digital simulation, Archwire, Tooth movement, Skeletal anchorage

## Abstract

**Background:**

The aim of this study is to compare the biomechanical effects of the conventional 0.019 × 0.025-in stainless steel archwire with the dual-section archwire when en-masse retraction is performed with sliding mechanics and skeletal anchorage.

**Methods:**

Models of maxillary dentition equipped with the 0.019 × 0.025-in archwire and the dual-section archwire, whose anterior portion is 0.021 × 0.025-in and posterior portion is 0.018 × 0.025-in were constructed. Then, long-term tooth movement during en-masse retraction was simulated using the finite element method. Power arms of 8, 10, 12 and 14 mm length were employed to control anterior torque, and retraction forces of 2 N were applied with a direct skeletal anchorage.

**Results:**

For achieving bodily movement of the incisors, power arms longer than 14 mm were required for the 0.019 × 0.025-in archwire, while between 8 and 10 mm for the dual-section archwire. The longer the power arms, the greater the counter-clockwise rotation of the occlusal plane was produced. Frictional resistance generated between the archwire and brackets and tubes on the posterior teeth was smaller than 5% of the retraction force of 2 N.

**Conclusions:**

The use of dual-section archwire might bring some biomechanical advantages as it allows to apply retraction force at a considerable lower height, and with a reduced occlusal plane rotation, compared to the conventional archwire. Clinical studies are needed to confirm the present results.

## Background

In extraction cases, retraction of the anterior teeth with orthodontic fixed appliances is often associated with several undesirable side effects such as deepening of the bite, rotation of the occlusal plane, archwire bowing and loss of posterior anchorage [1]. These effects, often related with the use of sliding mechanics, may not only extend treatment time but also compromise the outcomes [2–4]. Numerous techniques proved to be effective in overcoming undesirable side effects in extraction cases with sliding mechanics. Miniscrews can reinforce posterior anchorage [5], but they can’t avoid the loss of anterior torque control, nor prevent  bowing effect [1]. On the other hand, prescriptions featuring hyper-torque for the brackets of incisors may reduce an excessive lingual crown tipping during the retraction phase. However, bodily movements remain difficult to obtain using the undersized wires that are necessary to reduce friction unavoidably generated in sliding mechanics [6, 7]. Recent research on the effective size and shape of bracket slots has cast further doubt on the ability of pre-programmed appliances to provide effective torque control for the anterior teeth during space closure [8, 9].

Hence, based on biomechanical studies, several authors have suggested the use of power arms of different lengths depending on the desired type of anterior tooth movement. This would enable clinicians to achieve controlled tooth movement with fewer side effects during space closure in sliding mechanics [10]. On the other hand, Tominaga et al. [7] pointed out that it would be difficult to achieve bodily movement or root movement of the incisors by using power arms alone. This finding has recently been confirmed by a finite element (FE) study wherein long-term orthodontic tooth movement throughout the space closure phase was reproduced using a numerical simulation model [11]. Also, the use of extremely long power arms, which becomes necessary for improving controlled movements of the anterior teeth, might compromise patient comfort [7, 12].

**Fig. 1:**
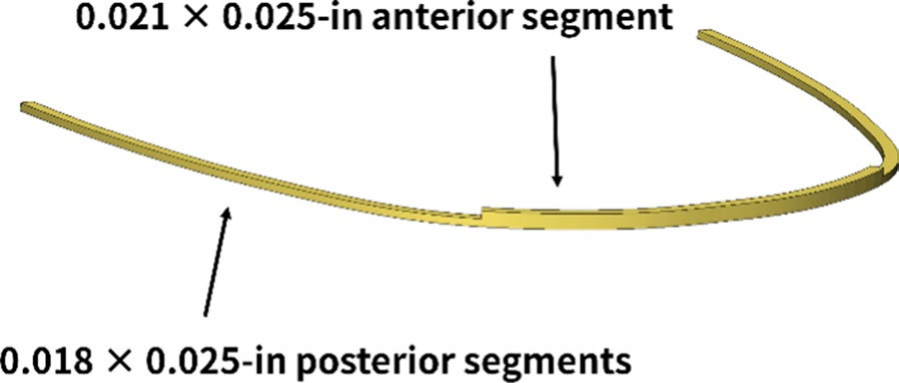
Dual-section archwire, whose cross section is 0.021 × 0.025-in in the anterior portion for increasing the incisor torque control, and 0.018 × 0.025-in in the posterior portion for reducing friction as compared to the 0.019 × 0.025-in archwire

Aiming to overcoming biomechanical limitations mentioned above, Cantarella et al. [13] have proposed the use of rectangular dual-section archwires for 0.022 bracket slot system (Fig. 1) whose cross section is 0.021 × 0.025-in in the anterior portion and 0.018 × 0.025-in in the posterior portion. This archwire was designed to maintain the friction low in the posterior sectors due to undersized cross section as compared to the conventional rectangular wire of 0.019 × 0.025-in. Conversely, the anterior portion of the archwire is thicker, thereby reducing the play between the archwire and brackets, and consequently providing a more effective torque control in the incisor region. However, its advantage over conventional wires with respect to the controlled movement of the incisors has not been quantified yet.

The purpose of this study was to evaluate the biomechanical effect of the dual-section archwires on the movement pattern of the anterior teeth during en-masse retraction with direct skeletal anchorage and to compare it with the conventional 0.019 × 0.025-in archwires. We constructed FE models with realistic bracket slot, archwire and tooth dimensions and simulated long-term tooth movement. We also determined the frictional resistance between the archwire and brackets in the course of time by means of the FE method, which can quantify and visualize long-term effect of orthodontic appliances. The null hypothesis is that the conventional 0.019 × 0.025-in and the dual-section archwires have the same biomechanical effect during en-masse retraction of anterior teeth when direct skeletal anchorage is employed.

## Methods

The method constructing a three-dimensional FE model of the maxillary dentition for simulating a long-term tooth movement and the material properties assigned to the elements were described in detail in a previous article [11]. This model allows to evaluate the force system acting on each tooth as well as tooth movement pattern in the course of treatment. The first premolar was removed to construct the model of an extraction case. Then, the extraction space was reduced to 4 mm on the assumption that the space was partly decreased during the initial leveling. Hundreds of steps for initial tooth displacements were iterated until the extraction space was closed. All FE analyses were performed using a FE solver software Marc 2017.1 (MSC Software Corp.).

**Fig. 2:**
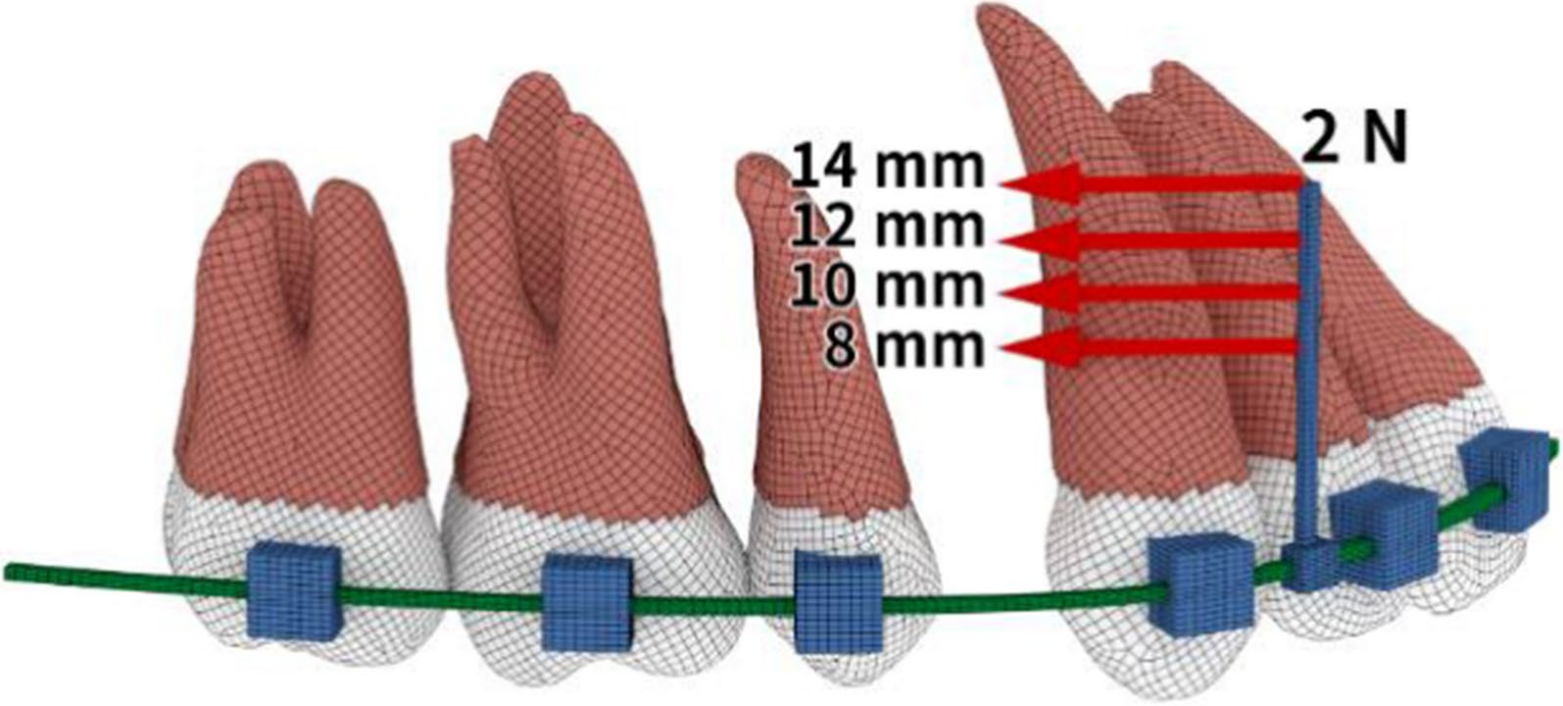
Loading conditions. A retraction force of 2 N was applied to the power arms parallel to the archwire. The height levels on the power arms were 8, 10, 12 and 14 mm from the bracket slot

0.022 × 0.028-in slot passive self-ligating brackets and stainless steel archwires with power arms were modeled. We tested two types of archwire, namely, conventional 0.019 × 0.025-in archwire and dual-section archwire with 0.021 × 0.025-in anterior segment and 0.018 × 0.025-in posterior segments (Fig. 1). Hence, the play between the archwire and brackets was precisely modeled. We also tested four types of power arms whose lengths were 8, 10, 12 and 14 mm (Fig. 2).

A retraction force of 2 N was applied to the top end of the power arm for the space closure. The force was directed toward a miniscrew on the assumption that it was placed between the second premolar and first molar on the buccal side as a direct skeletal anchorage. Miniscrews were located at the same level as the height of the power arm to provide a retraction force parallel to the archwire and the occlusal plane.

Tooth movement pattern was evaluated by using the ratio of the incisor’s lingual crown tipping (measured in degrees) to the displacement of the incisor’s center of resistance (CR) (measured in mm) in the palatal direction. The position of the CR of each tooth was determined according to the method proposed in a previous study [11]. The degree of rotation of the maxillary occlusal plane was measured using the line connecting the bracket slot on the central incisor and the tube on the first molar.

Contact boundary condition was set so that each tooth, bracket and archwire could contact each other. Frictional coefficient between the archwire and brackets was set to be 0.08 according to the previous studies [14–16]. Frictional resistance against sliding of the archwire was computed as the sum of the tangential frictional forces acting on the tubes of the molars and the bracket of the second premolar.

## Results

**Fig. 3:**
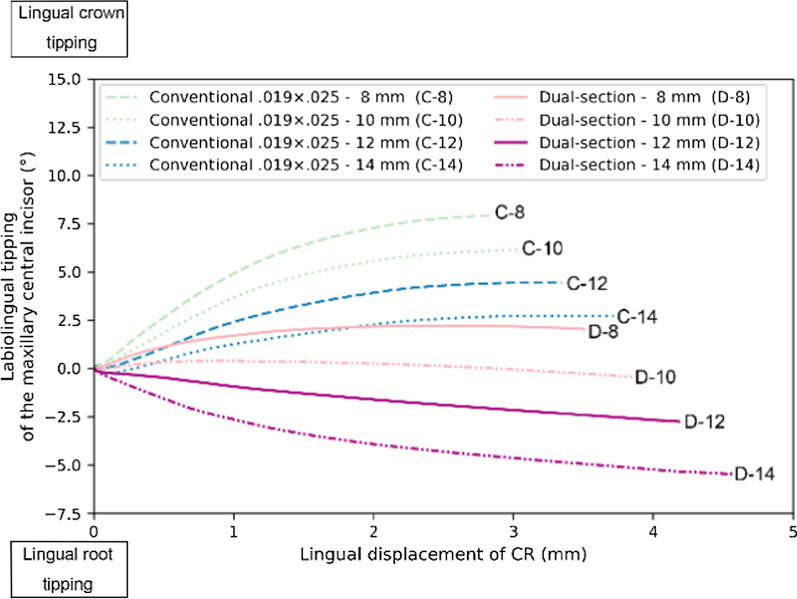
Degree of labiolingual tipping of the maxillary central incisor as a function of the amount of lingual displacement of the CR. Positive signs indicate lingual crown tipping and negative signs lingual root tipping. C-8 represents the conventional 0.019 × 0.025-in archwire with 8 mm power arm, and D-14 the dual-section archwire with 14 mm power arm, for instance

When the 0.019 × 0.025-in archwire was used in sliding mechanics with direct skeletal anchorage, the incisor showed significantly greater lingual crown tipping than when using the dual-section archwire (0.021 × 0.025-in anterior; 0.018 × 0.025-in posterior) as shown in Fig. 3. At the time of completion of the extraction space closure, the use of the 0.019 × 0.025-in archwire produced the incisor’s lingual crown tipping of 7.9, 6.2, 4.5 and 2.7 degrees, with the power arms of 8, 10, 12 and 14 mm, respectively. On the other hand, the employment of the dual-section archwire generated the incisor’s lingual crown tipping of 2.0 degrees when the power arm of 8 mm was used. As the length of power arm was increased from 8 mm, the direction of tooth rotation was changed from lingual crown tipping to lingual root tipping. Consequently, the incisor showed lingual root tipping of 0.4, 2.7 and 5.5 degrees using power arms of 10, 12 and 14 mm, respectively.

**Fig. 4:**
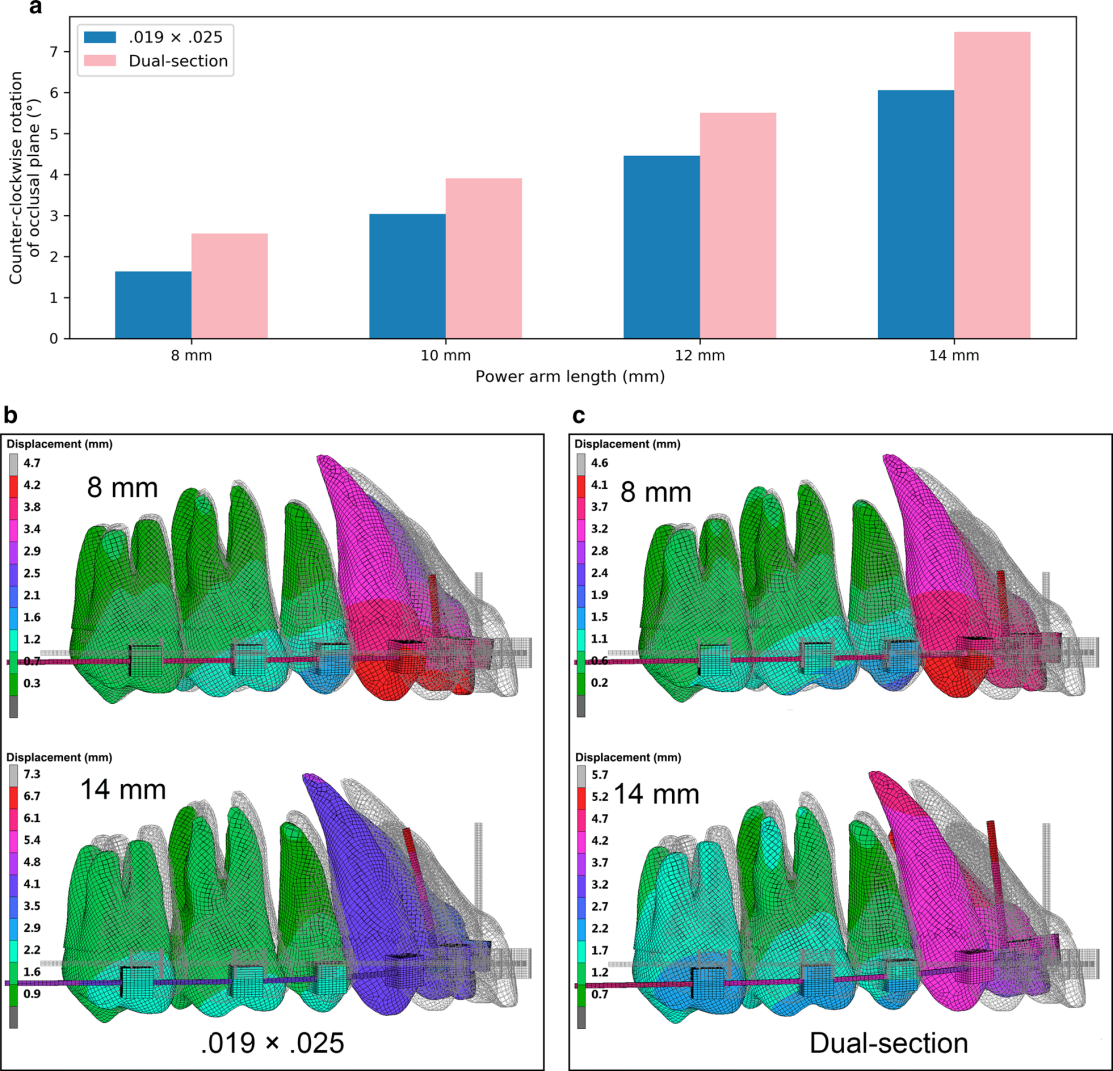
Degree of rotation of the occlusal plane as a function of power arm length and tooth movement patterns. **a** Degree of rotation of the occlusal plane with varying the height of retraction force on power arm from 8 to 14 mm at the completion of space closure for the 0.019 × 0.025-in and the dual-section archwire. **b** Tooth movement patterns with the 0.019 × 0.025-in archwire and power arms of 8 and 14 mm at the time when the extraction space was closed. Power arm of 14 mm was required to achieve bodily movement of the incisors. However, it caused a large amount of counter-clockwise rotation of the occlusal plane. **c** Tooth movement patterns with the dual-section archwire and power arms of 8 and 14 mm. Bodily movement of the incisors was produced with a small amount of counter-clockwise rotation of the occlusal plane when 8 mm power arm was used

The longer the power arm, the more substantially the occlusal plane was rotated (Fig. 4). At the time of completion of space closure, the use of the 0.019 × 0.025-in archwire produced counter-clockwise rotation of the occlusal plane of 1.6, 3.0, 4.5 and 6.1 degrees with the power arms of 8, 10, 12 and 14 mm, respectively. With the dual-section archwire, the occlusal plane was rotated a little more substantially than with the 0.019 × 0.025-in archwire. That is, degrees of rotation were 2.6, 3.9, 5.5 and 7.5 with the power arms of 8, 10, 12 and 14 mm, respectively.

**Fig. 5:**
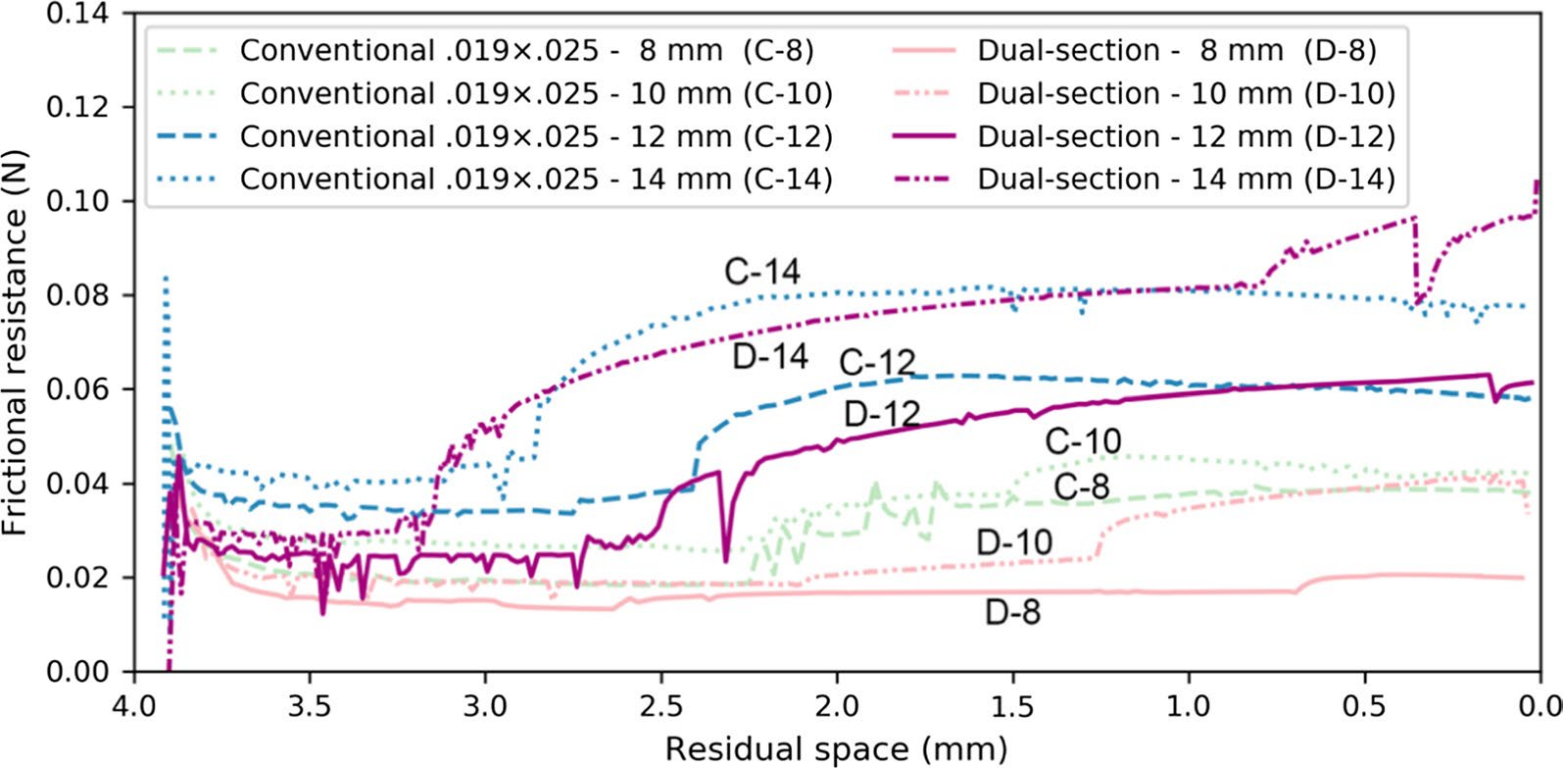
Frictional resistance generated at the interface between the archwire and the tubes and brackets on the posterior teeth with a 0.019 × 0.025-in archwire and dual-section archwire as a function of the remaining extraction space. Frictional resistance less than 0.1 N was observed in both cases

Figure 5 shows the sum of frictional resistance generated at the interface between the archwire and the tubes on all the posterior teeth. The amount of frictional resistance had a tendency to increase as the space was closed in both cases, which ranged from 0.01 to 0.1 N during space closure. Frictional resistance at the interface between the archwire and posterior brackets with the 0.019 × 0.025-in archwire was greater than that with the dual-section archwire up to the residual space of 1 mm, although no clear difference was found between them.

**Fig. 6:**
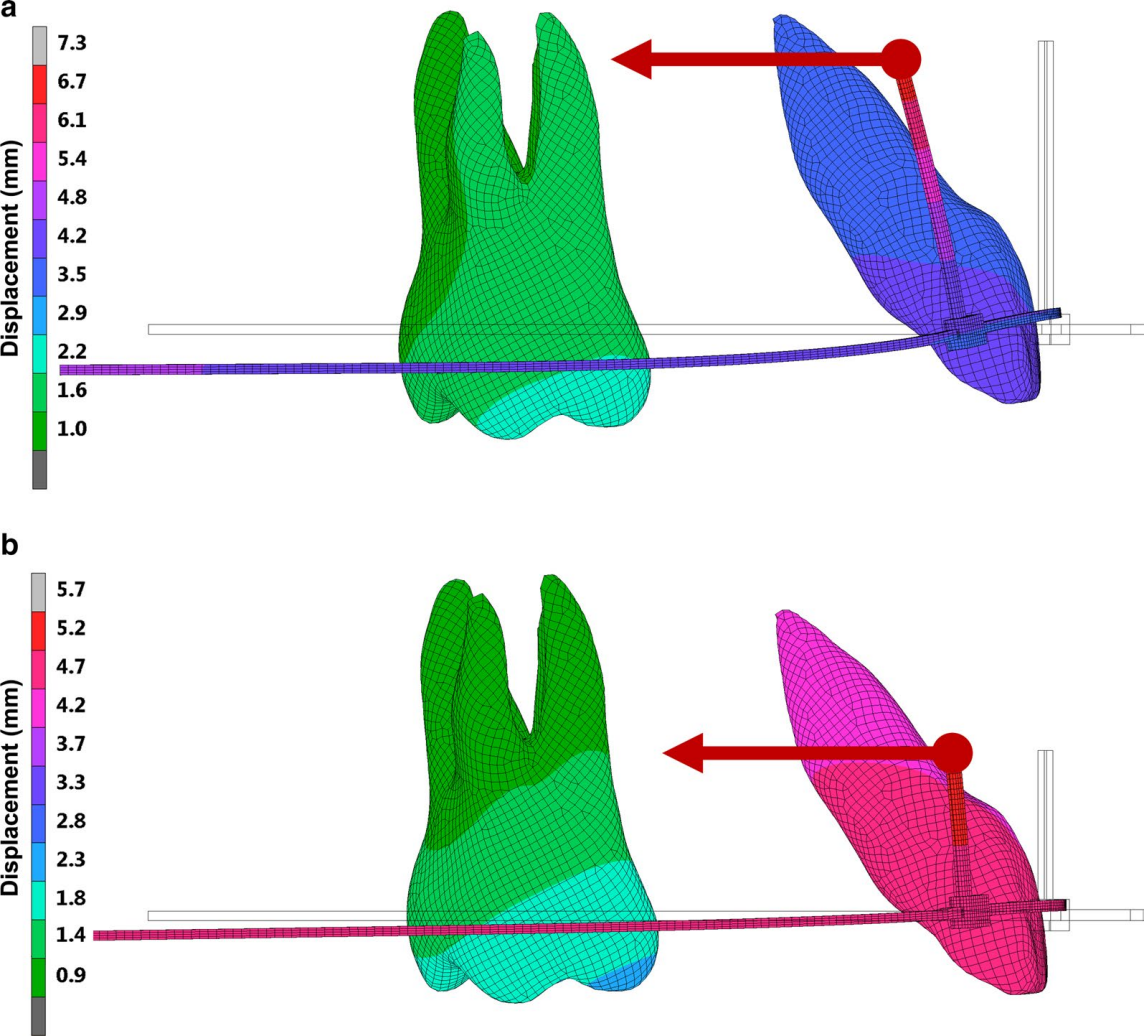
Deflection of the archwire at the completion of space closure when bodily movement of the incisor was produced. **a** 0.019 × 0.025-in archwire with power arm longer than 14 mm. **b** Dual-section archwire with power arm 8 mm long

Figure 6 shows the deflection of the archwire when bodily movement of the incisor was achieved at the time of completion of space closure. A larger amount of the archwire deflection was observed with the 0.019 × 0.025-in archwire than with the dual-section archwire. The anterior portion of the archwire was raised upward in both cases.

## Discussion

When incisors are retracted in sliding mechanics with direct skeletal anchorage, power arms have the effect of increasing the incisor torque expression. The present study showed that the use of power arms produced a deflection of the archwire (Fig. 6), so that its anterior portion is lifted in an apical direction and twisted in the third order of space. It was found that the longer the power arms, the more substantially the archwire could be deformed, and higher the incisor torque expression could be. Tominaga et al. [7] have also suggested that the anterior portion of the archwire is subjected to torsion caused by power arms, and consequently the torque is practically transmitted to the bracket on the incisor. The results obtained from this study suggested that when the conventional 0.019 × 0.025-in archwire is used, a power arm longer than 14 mm is required to achieve bodily movement of the central incisor because of the large bracket-archwire play. Such a long power arm would not be clinically applicable because it could cause patient discomfort. In addition, this length of power arm would require to place miniscrews in a very apical position, beyond the mucogingival junction, in the gingival mucosa, which is more prone to inflammation and where miniscrews stability is lower [17]. Furthermore, especially due to manufacturing tolerances, the play between the archwire and bracket is larger than the theoretically ideal play [8, 9], which may exacerbate this problem in the clinical practice.

Besides patient discomfort, the longer the power arm, the more substantially the occlusal plane is rotated counter-clockwise as an undesirable side effect (Fig. 4). Above-mentioned results support the previous study suggesting that a retraction force height superior to the CR of the entire maxillary dentition will cause the counter-clockwise rotation of the occlusal plane [1]. In the present analysis, the counter-clockwise rotation of the occlusal plane became minimal when the length of power arms was 8 mm (Fig. 4a). These results are in agreement with the finding that the CR of the FE model for the entire maxillary dentition, which was used in this study, was located at the level of 8.1 mm from the bracket slot. The counter-clockwise rotation of the maxillary occlusal plane and the resultant molar extrusion could cause clockwise rotation of the mandible, and consequently the facial profile would be worsened in the treatment of maxillary protrusion cases. This event would be particularly harmful in Class II dolichocephalic patients, where a clockwise rotation of the mandible further reduces the chin projection. Conversely, the utilization of power arms of approximately 8 mm would maintain the inclination of the occlusal plane during incisor retraction, and prevent unwanted clockwise mandibular rotation.

On the other hand, the dual-section archwire has shown some advantages as compared to the conventional 0.019 × 0.025-in archwire. The cross section of the anterior portion of the dual-section archwire is 0.021 × 0.025-in, which reduces the play between the archwire and brackets, thereby minimizing the loss of anterior torque control and increasing the torque expression in the bracket slots. As a result, the use of the dual-section archwire allows for achieving bodily movement of the incisors in combination with a shorter power arm than the 0.019 × 0.025-in archwire (Fig. 3). The present study indicates that the dual-section archwire could reduce power arm length to approximately 8 mm, which causes minimal counter-clockwise rotation of the maxillary occlusal plane, when bodily movement is required (Fig. 4).

Frictional resistance at the interface between the archwire and posterior brackets with the 0.019 × 0.025-in archwire was greater than that with the dual-section archwire up to the residual space of 1 mm, although there was no significant difference between them (Fig. 5). The values of frictional resistance ranged from 0.01 to 0.1 N, which were smaller than 5% of the retraction force of 2 N and seemed not to give great impact on tooth movement. This is because no external force was applied directly to the posterior brackets when a skeletal anchorage was employed.

The frictional resistance was found to be negligible in the present study. Nevertheless, a greater amount of frictional resistance could be generated in case of reciprocal retraction without using skeletal anchorage. Although the dual-section archwire is considered to have an advantage under such a situation, further investigations and clinical studies are needed to verify the biomechanical effectiveness of the dual-section archwire with respect to its feature to reduce the amount of friction in the posterior segment due to its undersized cross section.

Most of studies on the simulation of orthodontic tooth movement had been limited to analyses of the initial displacement [6, 10, 12]. It was therefore difficult to precisely predict overall tooth movement, since it reflects the archwire deformation including torsion within the brackets, which could change the force system during space closure, thereby exerting great influence on the torquing effect and the resultant incisor movement. The novel method employed in the present study enabled us to predict the long-term orthodontic tooth movement and to accurately determine the force system acting on each tooth in the course of treatment. As a result, biomechanical effects of the conventional 0.019 × 0.025-in and dual-dimension archwires on the anterior tooth movement could be successfully evaluated and compared. The null hypothesis was rejected; the dual-section archwires produced a more favorable biomechanical effect, compared to the conventional 0.019 × 0.025-in archwires, during en-masse retraction of anterior teeth with the use of direct skeletal anchorage.

One limitation of this study is its computational nature. These results should be confirmed by clinical trials.

## Conclusions

- When using the 0.019 × 0.025-in archwire, power arms longer than 14 mm are necessary to achieve bodily movement of the incisors, causing a large amount of counter-clockwise rotation of the occlusal plane.
- When the dual-section archwire (0.021 × 0.025-in anterior; 0.018 × 0.025-in posterior) is used, the power arm length could be reduced to approximately 8 mm for achieving bodily movement with a minimal rotation of the occlusal plane.
- Simulation of long-term tooth movement using the FE method is considered to be a suitable and useful tool to analyze the tooth movement pattern, force system acting on each tooth, and side effects that take place during orthodontic treatment.

## Data Availability

Data of the present study will be shared upon request to the corresponding Author.

## Abbreviations

IN: Inches
FE: Finite element
